# Supplementary material for: Genome-wide DNA methylation analysis of heavy cannabis exposure in a New Zealand longitudinal cohort
Source: Transl Psychiatry. 2020 Apr 22;10:114. doi: 10.1038/s41398-020-0800-3 (PMC7176736; doi:10.1038/s41398-020-0800-3)
Supplement: Supplementary file 1 — Supplementary Material [file 41398_2020_800_MOESM1_ESM.docx]

**Supplementary material**

Genome-wide DNA methylation analysis of heavy cannabis exposure in a New Zealand longitudinal cohort.

Amy J. Osborne,^1,6^* John F. Pearson,^2,6^ Alexandra J. Noble,^1^ Neil J. Gemmell,^3^ L. John Horwood,^4^ Joseph M. Boden,^4^ Miles Benton,^5^ Donia P. Macartney-Coxson,^5^ Martin A. Kennedy^2^**

|  |  | **Cases** | **Controls** |
| --- | --- | --- | --- |
| Sex | Male | 37 | 37 |
|  | Female | 11 | 11 |
| Ethnicity | European | 35 | 45 |
|  | Other | 13 | 3 |
| Socioeconomic status | Professional/managerial | 6 | 6 |
|  | Clerical/technical/skilled | 21 | 21 |
|  | Semi-skilled/unskilled | 21 | 21 |

**Supplementary Table 1.**  Christchurch Health and Development Study (CHDS) participants selected for EPIC arrays. Cases and controls were matched as closely as possible by sex, ethnicity and parental socioeconomic status/occupation. Cases are comprised of regular cannabis users, half of whom have never used tobacco. Controls are comprised of individuals with no exposure to cannabis or tobacco. ‘Other’ ethnicity is a combination of Māori and Pacific Island participants.

|  | **Multiple methylation sites** | |
| --- | --- | --- |
|  | hypermethylated | hypomethylated |
| TP73 | 8 |  |
| DNAH1 | 4 |  |
| TMEM190 | 4 |  |
| DNALI1 | 3 |  |
| MYO1G | 3 |  |
| VSTM1 | 3 |  |
| ARHGAP27 | 2 |  |
| ARHGEF1 | 2 |  |
| DBI | 2 |  |
| DGKD | 2 |  |
| DUS3L | 2 |  |
| FBRSL1 | 1 | 1 |
| GAS7 | 2 |  |
| GATD3A | 2 |  |
| KLHDC4 | 2 |  |
| KRTAP2-1 | 2 |  |
| LTBP3 | 2 |  |
| MIR7-2 | 2 |  |
| PRKN | 2 |  |
| PSMB2 | 2 |  |
| PTCHD3P1 | 2 |  |
| PTCHD4 | 2 |  |
| RAD51B |  | 2 |
| RPS6KA2 | 2 |  |
| SORL1 | 1 | 1 |
| STEAP3 | 2 |  |
| UBE2E2 | 2 |  |
| ZDHHC1 | 2 |  |

**Supplementary Table 2** – Genes with multiple nominally-significant CpG sites in cannabis-only participants.

**Supplementary Table 3 -** Differentially methylated loci in cannabis-only participants at a nominal P<0.001.

| **Illumina ID** | **effect** | **P Value** | **P value** | **GENEID** | **Location** | **Distance** | **up/down** | **in/out** |
| --- | --- | --- | --- | --- | --- | --- | --- | --- |
| cg00470351 | 0.02377 | 0.00001 | 0.629 | CDC20 | inside exon | 517 | hyper | in |
| cg06693983 | 0.08083 | 0.00001 | 0.688 | TMEM190 | inside exon | 1012 | hyper | in |
| cg26069230 | 0.01245 | 0.00001 | 0.688 | ADAP2 | inside exon | 1319 | hyper | in |
| cg25293806 | 0.05848 | 0.00002 | 0.776 | TMEM190 | inside exon | 1183 | hyper | in |
| cg09221413 | 0.02661 | 0.00002 | 0.776 | FKBP10 | inside exon | 290 | hyper | in |
| cg07178825 | 0.03047 | 0.00003 | 0.880 | TP73 | inside exon | 5532 | hyper | in |
| cg03938432 | 0.01322 | 0.00004 | 0.885 | ADGRB3 | inside exon | 204 | hyper | in |
| cg22030419 | 0.00376 | 0.00006 | 0.985 | ZNF416 | inside exon | 21 | hyper | in |
| cg10162691 | 0.00821 | 0.00009 | 0.985 | RND2 | inside exon | 151 | hyper | in |
| cg10098021 | 0.00621 | 0.00009 | 0.985 | PPP1R18 | inside exon | 1940 | hyper | in |
| cg00345862 | 0.01019 | 0.00009 | 0.985 | CNMD | inside exon | 102 | hyper | in |
| cg22897141 | 0.05364 | 0.00010 | 0.985 | MEN1 | inside exon | 1428 | hyper | in |
| cg22887000 | 0.05880 | 0.00014 | 0.985 | VSTM1 | inside exon | 147 | hyper | in |
| cg27390009 | 0.02987 | 0.00015 | 0.985 | FLII | inside exon | 11729 | hyper | in |
| cg27608102 | 0.02861 | 0.00015 | 0.985 | KRT19 | inside exon | 4137 | hyper | in |
| cg08941759 | 0.00354 | 0.00017 | 0.985 | CHCHD6 | inside exon | 24 | hyper | in |
| cg06044455 | 0.02146 | 0.00020 | 0.985 | SNED1 | inside exon | 17664 | hyper | in |
| cg15722151 | 0.01059 | 0.00021 | 0.985 | RTBDN | inside exon | 11 | hyper | in |
| cg00859792 | 0.02072 | 0.00021 | 0.985 | GCNT2 | inside exon | 120 | hyper | in |
| cg24716530 | 0.03871 | 0.00022 | 0.985 | LTBP3 | inside exon | 4911 | hyper | in |
| cg20371891 | 0.01001 | 0.00022 | 0.985 | RAB20 | inside exon | 319 | hyper | in |
| cg06108383 | 0.01947 | 0.00023 | 0.985 | LOC100507547 | inside exon | 1243 | hyper | in |
| cg17804348 | 0.08234 | 0.00026 | 0.985 | TP73 | inside exon | 5431 | hyper | in |
| cg16343401 | 0.03726 | 0.00028 | 0.985 | GFAP | inside exon | 3773 | hyper | in |
| cg23793397 | 0.01091 | 0.00028 | 0.985 | EPOP | inside exon | 1429 | hyper | in |
| cg01155426 | 0.02213 | 0.00028 | 0.985 | PSMB2 | inside exon | 68022 | hyper | in |
| cg04652097 | 0.02091 | 0.00029 | 0.985 | MAGI2-AS3 | inside exon | 169 | hyper | in |
| cg14700841 | 0.00692 | 0.00030 | 0.985 | TRIM27 | inside exon | 20392 | hyper | in |
| cg13263950 | 0.01972 | 0.00031 | 0.985 | PSMB2 | inside exon | 68049 | hyper | in |
| cg05619414 | 0.01576 | 0.00033 | 0.985 | XPNPEP3 | inside exon | 52049 | hyper | in |
| cg05333146 | 0.01459 | 0.00033 | 0.985 | B3GNT9 | inside exon | 1177 | hyper | in |
| cg22392708 | 0.01020 | 0.00033 | 0.985 | KRT23 | inside exon | 66 | hyper | in |
| cg12282552 | 0.01494 | 0.00039 | 0.985 | LTBP3 | inside exon | 4447 | hyper | in |
| cg00966482 | 0.02640 | 0.00039 | 0.985 | ERVFRD-1 | inside exon | 145 | hyper | in |
| cg03540165 | 0.00663 | 0.00040 | 0.985 | RASAL1 | inside exon | 337 | hyper | in |
| cg03048314 | 0.02950 | 0.00042 | 0.985 | NBL1 | inside exon | 129 | hyper | in |
| cg08758850 | 0.00478 | 0.00042 | 0.985 | NR6A1 | inside exon | 22 | hyper | in |
| cg04422168 | 0.01322 | 0.00045 | 0.985 | PTPRN | inside exon | 13 | hyper | in |
| cg17452880 | 0.00576 | 0.00045 | 0.985 | NUAK1 | inside exon | 72974 | hyper | in |
| cg02172711 | 0.03132 | 0.00046 | 0.985 | PATL2 | inside exon | 4464 | hyper | in |
| cg19971650 | 0.01088 | 0.00052 | 0.985 | GABRB2 | inside exon | 52 | hyper | in |
| cg25899024 | 0.01533 | 0.00055 | 0.985 | DBI | inside exon | 107 | hyper | in |
| cg16051421 | 0.01140 | 0.00055 | 0.985 | PML | inside exon | 11227 | hyper | in |
| cg20123729 | 0.00413 | 0.00056 | 0.985 | GFER | inside exon | 218 | hyper | in |
| cg06487289 | 0.01145 | 0.00059 | 0.985 | CHST2 | inside exon | 2568 | hyper | in |
| cg15030849 | 0.02569 | 0.00059 | 0.985 | FAM71E2 | inside exon | 8526 | hyper | in |
| cg21488617 | 0.01607 | 0.00059 | 0.985 | DNALI1 | inside exon | 66 | hyper | in |
| cg09731694 | 0.01541 | 0.00060 | 0.985 | C9orf50 | inside exon | 52 | hyper | in |
| cg15635600 | 0.02898 | 0.00060 | 0.985 | DNMT3L | inside exon | 265 | hyper | in |
| cg03743205 | 0.01764 | 0.00066 | 0.985 | ZFPM1 | inside exon | 80821 | hyper | in |
| cg21171625 | 0.01784 | 0.00066 | 0.985 | DNALI1 | inside exon | 87 | hyper | in |
| cg24969431 | 0.01397 | 0.00073 | 0.985 | TDRD5 | inside exon | 108 | hyper | in |
| cg03802998 | 0.01240 | 0.00074 | 0.985 | TP53I11 | inside exon | 113 | hyper | in |
| cg19402371 | 0.03487 | 0.00074 | 0.985 | TMEM190 | inside exon | 809 | hyper | in |
| cg13718185 | 0.02197 | 0.00074 | 0.985 | RHOBTB2 | inside exon | 20183 | hyper | in |
| cg16602500 | 0.03707 | 0.00075 | 0.985 | TUT4 | inside exon | 129187 | hyper | in |
| cg02924487 | 0.02714 | 0.00075 | 0.985 | TP73 | inside exon | 5302 | hyper | in |
| cg02386403 | 0.01316 | 0.00076 | 0.985 | FOXF2 | inside exon | 929 | hyper | in |
| cg25010998 | 0.00544 | 0.00076 | 0.985 | CAPNS1 | inside exon | 112 | hyper | in |
| cg21214758 | 0.00649 | 0.00081 | 0.985 | WASHC5 | inside exon | 81 | hyper | in |
| cg14532498 | 0.00592 | 0.00082 | 0.985 | HRAT92 | inside exon | 2147 | hyper | in |
| cg04018474 | 0.01494 | 0.00083 | 0.985 | STX1B | inside exon | 13826 | hyper | in |
| cg09989847 | 0.03306 | 0.00085 | 0.985 | NT5E | inside exon | 45734 | hyper | in |
| cg14630801 | 0.03884 | 0.00085 | 0.985 | FRMD4A | inside exon | 131988 | hyper | in |
| cg03501901 | 0.01123 | 0.00085 | 0.985 | ESPN | inside exon | 12674 | hyper | in |
| cg06355010 | 0.00682 | 0.00087 | 0.985 | ISM1 | inside exon | 71 | hyper | in |
| cg02038852 | 0.03040 | 0.00087 | 0.985 | SLC7A10 | inside exon | 251 | hyper | in |
| cg17783317 | 0.04148 | 0.00089 | 0.985 | VSTM1 | inside exon | 84 | hyper | in |
| cg14036830 | 0.01439 | 0.00093 | 0.985 | GRIK5 | inside exon | 66750 | hyper | in |
| cg11664903 | 0.02375 | 0.00095 | 0.985 | KIRREL3-AS3 | inside exon | 3039 | hyper | in |
| cg09096555 | 0.03444 | 0.00096 | 0.985 | GRIN2C | inside exon | 8608 | hyper | in |
| cg19234858 | 0.00017 | 0.00097 | 0.985 | AUP1 | inside exon | 602 | hyper | in |
| cg21919809 | 0.00655 | 0.00097 | 0.985 | ETV2 | inside exon | 195 | hyper | in |
| cg03937116 | 0.01236 | 0.00098 | 0.985 | PI4KB | inside exon | 33223 | hyper | in |
| cg02345493 | 0.00268 | 0.00098 | 0.985 | PTTG1IP | inside exon | 166 | hyper | in |
| cg15925718 | 0.00496 | 0.00098 | 0.985 | ADK | inside exon | 89 | hyper | in |
| cg23927367 | 0.00567 | 0.00099 | 0.985 | THYN1 | inside exon | 458 | hyper | in |
| cg26896762 | 0.01062 | 0.00099 | 0.985 | ISL1 | inside exon | 403 | hyper | in |
| cg12803068 | 0.09514 | 0.00000 | 0.385 | MYO1G | inside intron | 15785 | hyper | in |
| cg02234936 | 0.01209 | 0.00000 | 0.385 | ARHGEF1 | inside intron | 20615 | hyper | in |
| cg01695406 | 0.04914 | 0.00000 | 0.629 | TMEM190 | inside intron | 1072 | hyper | in |
| cg24875484 | 0.00956 | 0.00000 | 0.629 | MUCL3 | inside intron | 1806 | hyper | in |
| cg05009104 | 0.04998 | 0.00001 | 0.629 | MYO1G | inside intron | 15724 | hyper | in |
| cg04180046 | 0.04471 | 0.00001 | 0.688 | MYO1G | inside intron | 15968 | hyper | in |
| cg00241731 | 0.07676 | 0.00002 | 0.776 | MAP9 | inside intron | 22927 | hyper | in |
| cg01538731 | 0.06797 | 0.00003 | 0.776 | KLHDC4 | inside intron | 38628 | hyper | in |
| cg26396680 | 0.04000 | 0.00003 | 0.880 | PPP1R32 | inside intron | 7822 | hyper | in |
| cg02624701 | 0.05398 | 0.00004 | 0.885 | SLC17A7 | inside intron | 4395 | hyper | in |
| cg00699993 | 0.01021 | 0.00004 | 0.885 | GRIA2 | inside intron | 275 | hyper | in |
| cg04830098 | 0.01096 | 0.00004 | 0.885 | GLS2 | inside intron | 620 | hyper | in |
| cg20925841 | 0.00755 | 0.00004 | 0.885 | INHBB | inside intron | 681 | hyper | in |
| cg11267549 | 0.05111 | 0.00006 | 0.985 | APBB2 | inside intron | 67498 | hyper | in |
| cg27060391 | 0.02827 | 0.00006 | 0.985 | ARHGEF1 | inside intron | 17286 | hyper | in |
| cg06239618 | 0.01060 | 0.00007 | 0.985 | TBXAS1 | inside intron | 173343 | hyper | in |
| cg13139972 | 0.01375 | 0.00009 | 0.985 | DKK2 | inside intron | 247416 | hyper | in |
| cg17163168 | 0.04267 | 0.00009 | 0.985 | TP73 | inside intron | 31606 | hyper | in |
| cg24654681 | 0.03069 | 0.00010 | 0.985 | NOP53 | inside intron | 14441 | hyper | in |
| cg05830220 | 0.05425 | 0.00010 | 0.985 | KLHDC4 | inside intron | 38613 | hyper | in |
| cg04079760 | 0.03243 | 0.00011 | 0.985 | ESRP2 | inside intron | 442 | hyper | in |
| cg20781438 | 0.00828 | 0.00011 | 0.985 | MACF1 | inside intron | 78211 | hyper | in |
| cg19092096 | 0.01280 | 0.00013 | 0.985 | ALX3 | inside intron | 848 | hyper | in |
| cg24688471 | 0.03214 | 0.00013 | 0.985 | MMEL1 | inside intron | 21666 | hyper | in |
| cg05413199 | 0.02078 | 0.00014 | 0.985 | ZDHHC1 | inside intron | 19885 | hyper | in |
| cg16794070 | 0.01327 | 0.00014 | 0.985 | PARD6A | inside intron | 414 | hyper | in |
| cg00141479 | 0.00837 | 0.00014 | 0.985 | PIP4K2A | inside intron | 411 | hyper | in |
| cg17192115 | 0.01433 | 0.00015 | 0.985 | GAS7 | inside intron | 238968 | hyper | in |
| cg20768358 | 0.07349 | 0.00015 | 0.985 | TP73 | inside intron | 5870 | hyper | in |
| cg13721989 | 0.00731 | 0.00015 | 0.985 | PIP5KL1 | inside intron | 3823 | hyper | in |
| cg19359071 | 0.01118 | 0.00016 | 0.985 | PRDM2 | inside intron | 3275 | hyper | in |
| cg09816507 | 0.01932 | 0.00017 | 0.985 | PTCHD4 | inside intron | 42359 | hyper | in |
| cg07099839 | 0.02512 | 0.00018 | 0.985 | FGD6 | inside intron | 47839 | hyper | in |
| cg11041018 | 0.00649 | 0.00018 | 0.985 | PLCB1 | inside intron | 177595 | hyper | in |
| cg00752478 | 0.01505 | 0.00018 | 0.985 | ZDHHC1 | inside intron | 19980 | hyper | in |
| cg09618848 | 0.01527 | 0.00019 | 0.985 | KCTD1 | inside intron | 148402 | hyper | in |
| cg13476950 | 0.03488 | 0.00020 | 0.985 | DNAH1 | inside intron | 1345 | hyper | in |
| cg16761329 | 0.04430 | 0.00020 | 0.985 | C5orf66 | inside intron | 9143 | hyper | in |
| cg04665374 | 0.02084 | 0.00020 | 0.985 | ITGA7 | inside intron | 5011 | hyper | in |
| cg25069388 | 0.03908 | 0.00020 | 0.985 | CSNK2A1 | inside intron | 26476 | hyper | in |
| cg10443049 | 0.01969 | 0.00020 | 0.985 | NXPH1 | inside intron | 7952 | hyper | in |
| cg02449166 | 0.01562 | 0.00021 | 0.985 | EBF3 | inside intron | 13610 | hyper | in |
| cg04608239 | 0.01244 | 0.00021 | 0.985 | GATD3A | inside intron | 9036 | hyper | in |
| cg27076669 | 0.01351 | 0.00022 | 0.985 | CPM | inside intron | 29563 | hyper | in |
| cg02355924 | 0.04676 | 0.00023 | 0.985 | Mar-04 | inside intron | 68283 | hyper | in |
| cg06202149 | 0.01412 | 0.00023 | 0.985 | TP73 | inside intron | 36757 | hyper | in |
| cg27405731 | 0.03096 | 0.00023 | 0.985 | CUX1 | inside intron | 110321 | hyper | in |
| cg21610871 | 0.04520 | 0.00023 | 0.985 | CACNA2D4 | inside intron | 73853 | hyper | in |
| cg01854076 | 0.02166 | 0.00024 | 0.985 | NAT8L | inside intron | 1797 | hyper | in |
| cg24239518 | 0.00573 | 0.00024 | 0.985 | CDH23 | inside intron | 198416 | hyper | in |
| cg11575865 | 0.01309 | 0.00025 | 0.985 | SFMBT2 | inside intron | 32493 | hyper | in |
| cg25799458 | 0.01202 | 0.00025 | 0.985 | MTMR14 | inside intron | 9557 | hyper | in |
| cg03824573 | 0.01115 | 0.00025 | 0.985 | CACNA1C | inside intron | 153377 | hyper | in |
| cg27250236 | 0.04159 | 0.00026 | 0.985 | PTCHD4 | inside intron | 42326 | hyper | in |
| cg22494035 | 0.01807 | 0.00026 | 0.985 | CCDC33 | inside intron | 9189 | hyper | in |
| cg10717148 | 0.00370 | 0.00027 | 0.985 | TSKU | inside intron | 747 | hyper | in |
| cg20910202 | 0.02930 | 0.00027 | 0.985 | KBTBD11 | inside intron | 766 | hyper | in |
| cg21247398 | 0.02657 | 0.00027 | 0.985 | PRKAG2-AS1 | inside intron | 1150 | hyper | in |
| cg13017471 | 0.02039 | 0.00027 | 0.985 | FNDC3B | inside intron | 73964 | hyper | in |
| cg27364231 | 0.01393 | 0.00027 | 0.985 | ESAM | inside intron | 3294 | hyper | in |
| cg17060249 | 0.00622 | 0.00027 | 0.985 | CIDEC | inside intron | 38585 | hyper | in |
| cg05138148 | 0.01394 | 0.00027 | 0.985 | TRABD2B | inside intron | 12667 | hyper | in |
| cg03994942 | 0.01991 | 0.00028 | 0.985 | LARGE2 | inside intron | 783 | hyper | in |
| cg18545449 | 0.04833 | 0.00029 | 0.985 | SLC39A11 | inside intron | 81207 | hyper | in |
| cg11857646 | 0.01698 | 0.00032 | 0.985 | KCNH2 | inside intron | 30771 | hyper | in |
| cg18650670 | 0.01162 | 0.00033 | 0.985 | IGDCC4 | inside intron | 26258 | hyper | in |
| cg22599707 | 0.01418 | 0.00034 | 0.985 | CCDC69 | inside intron | 9348 | hyper | in |
| cg22482215 | 0.01016 | 0.00035 | 0.985 | CASP8AP2 | inside intron | 574 | hyper | in |
| cg23295886 | 0.01842 | 0.00036 | 0.985 | PTPRM | inside intron | 420385 | hyper | in |
| cg13742305 | 0.01072 | 0.00037 | 0.985 | CASKIN1 | inside intron | 1850 | hyper | in |
| cg05845767 | 0.01487 | 0.00038 | 0.985 | CDH22 | inside intron | 30765 | hyper | in |
| cg26739532 | 0.01109 | 0.00039 | 0.985 | SYBU | inside intron | 46321 | hyper | in |
| cg10313047 | 0.04843 | 0.00039 | 0.985 | DNAH1 | inside intron | 1020 | hyper | in |
| cg08325845 | 0.01774 | 0.00039 | 0.985 | ZNRD1ASP | inside intron | 54075 | hyper | in |
| cg10084993 | 0.01796 | 0.00040 | 0.985 | SLC9A3R2 | inside intron | 778 | hyper | in |
| cg10956403 | 0.04336 | 0.00040 | 0.985 | SPAG9 | inside intron | 8022 | hyper | in |
| cg04570735 | 0.01222 | 0.00041 | 0.985 | PDE4C | inside intron | 9361 | hyper | in |
| cg03302268 | 0.03886 | 0.00041 | 0.985 | TARM1 | inside intron | 4965 | hyper | in |
| cg06671242 | 0.02690 | 0.00042 | 0.985 | PRSS23 | inside intron | 16276 | hyper | in |
| cg23928920 | 0.05162 | 0.00044 | 0.985 | ST8SIA2 | inside intron | 7616 | hyper | in |
| cg20991988 | 0.02800 | 0.00045 | 0.985 | CREB5 | inside intron | 7767 | hyper | in |
| cg09765310 | 0.02113 | 0.00045 | 0.985 | TRPM2 | inside intron | 6978 | hyper | in |
| cg27302296 | 0.01198 | 0.00045 | 0.985 | TRIQK | inside intron | 51580 | hyper | in |
| cg04412755 | 0.04295 | 0.00046 | 0.985 | PTH1R | inside intron | 16239 | hyper | in |
| cg03107955 | 0.01107 | 0.00047 | 0.985 | LOC283177 | inside intron | 65769 | hyper | in |
| cg21629500 | 0.00781 | 0.00048 | 0.985 | C5orf38 | inside intron | 2491 | hyper | in |
| cg22295879 | 0.00338 | 0.00048 | 0.985 | PRIM1 | inside intron | 278 | hyper | in |
| cg23703946 | 0.03663 | 0.00048 | 0.985 | SLC8A1 | inside intron | 293621 | hyper | in |
| cg03128361 | 0.00975 | 0.00050 | 0.985 | DLEU1 | inside intron | 148648 | hyper | in |
| cg11936362 | 0.00748 | 0.00051 | 0.985 | PRKN | inside intron | 124210 | hyper | in |
| cg13136639 | 0.01748 | 0.00052 | 0.985 | ARHGAP27 | inside intron | 8439 | hyper | in |
| cg15507160 | 0.00613 | 0.00053 | 0.985 | ZNF487 | inside intron | 25181 | hyper | in |
| cg26105278 | 0.02536 | 0.00054 | 0.985 | TP73 | inside intron | 9444 | hyper | in |
| cg09839635 | 0.02584 | 0.00054 | 0.985 | RNF180 | inside intron | 132 | hyper | in |
| cg12606911 | 0.01321 | 0.00054 | 0.985 | RMND5A | inside intron | 89212 | hyper | in |
| cg04887826 | 0.02082 | 0.00056 | 0.985 | BOLA2 | inside intron | 506980 | hyper | in |
| cg02656472 | 0.05377 | 0.00056 | 0.985 | ITGB3 | inside intron | 69428 | hyper | in |
| cg13425938 | 0.01245 | 0.00057 | 0.985 | EGFL7 | inside intron | 2173 | hyper | in |
| cg01456368 | 0.04735 | 0.00057 | 0.985 | DNAH1 | inside intron | 1524 | hyper | in |
| cg09207494 | 0.00340 | 0.00057 | 0.985 | FNTB | inside intron | 357 | hyper | in |
| cg15215033 | 0.02618 | 0.00058 | 0.985 | SLC38A10 | inside intron | 10364 | hyper | in |
| cg03198578 | 0.02103 | 0.00059 | 0.985 | TMC4 | inside intron | 8340 | hyper | in |
| cg18913076 | 0.00439 | 0.00059 | 0.985 | MRPL30 | inside intron | 13059 | hyper | in |
| cg27505260 | 0.01865 | 0.00059 | 0.985 | GATD3A | inside intron | 10555 | hyper | in |
| cg02017733 | 0.02204 | 0.00060 | 0.985 | PISD | inside intron | 40244 | hyper | in |
| cg26055950 | 0.03376 | 0.00062 | 0.985 | TP73 | inside intron | 31723 | hyper | in |
| cg01146063 | 0.02014 | 0.00062 | 0.985 | CTNNA2 | inside intron | 17731 | hyper | in |
| cg23093164 | 0.05710 | 0.00062 | 0.985 | RCAN1 | inside intron | 87914 | hyper | in |
| cg04593523 | 0.04145 | 0.00062 | 0.985 | ETV6 | inside intron | 109415 | hyper | in |
| cg19013611 | 0.03433 | 0.00063 | 0.985 | PTPRN2 | inside intron | 712431 | hyper | in |
| cg16935813 | 0.00798 | 0.00063 | 0.985 | MXI1 | inside intron | 14742 | hyper | in |
| cg10872212 | 0.01547 | 0.00063 | 0.985 | KLHDC8B | inside intron | 142 | hyper | in |
| cg11198596 | 0.03691 | 0.00064 | 0.985 | LRIG1 | inside intron | 2728 | hyper | in |
| cg12954529 | 0.02063 | 0.00065 | 0.985 | NRG1 | inside intron | 41034 | hyper | in |
| cg02045669 | 0.03155 | 0.00066 | 0.985 | MAPK10 | inside intron | 357 | hyper | in |
| cg13886338 | 0.02323 | 0.00068 | 0.985 | MROH6 | inside intron | 4697 | hyper | in |
| cg24013620 | 0.04034 | 0.00068 | 0.985 | MAPK15 | inside intron | 181 | hyper | in |
| cg13537567 | 0.03618 | 0.00072 | 0.985 | CPLX2 | inside intron | 19960 | hyper | in |
| cg04651153 | 0.01207 | 0.00073 | 0.985 | GAS7 | inside intron | 145240 | hyper | in |
| cg00665971 | 0.01564 | 0.00073 | 0.985 | HRK | inside intron | 1462 | hyper | in |
| cg02254056 | 0.00924 | 0.00073 | 0.985 | BLOC1S5-TXNDC5 | inside intron | 153167 | hyper | in |
| cg15970457 | 0.00631 | 0.00073 | 0.985 | CTBP2 | inside intron | 56871 | hyper | in |
| cg11407215 | 0.00487 | 0.00074 | 0.985 | UXS1 | inside intron | 649 | hyper | in |
| cg18037921 | 0.02889 | 0.00075 | 0.985 | ALDH1A3 | inside intron | 19470 | hyper | in |
| cg07372166 | 0.03709 | 0.00075 | 0.985 | PRUNE2 | inside intron | 107337 | hyper | in |
| cg12749467 | 0.02389 | 0.00075 | 0.985 | BABAM2 | inside intron | 398414 | hyper | in |
| cg11782635 | 0.01475 | 0.00076 | 0.985 | KCNIP1 | inside intron | 150038 | hyper | in |
| cg00433654 | 0.01314 | 0.00076 | 0.985 | INO80B | inside intron | 2110 | hyper | in |
| cg03238513 | 0.02191 | 0.00078 | 0.985 | GPATCH2L | inside intron | 1135 | hyper | in |
| cg26661889 | 0.02281 | 0.00078 | 0.985 | MN1 | inside intron | 8734 | hyper | in |
| cg05355157 | 0.01103 | 0.00079 | 0.985 | C4BPB | inside intron | 5196 | hyper | in |
| cg10658345 | 0.04728 | 0.00079 | 0.985 | CLPX | inside intron | 2601 | hyper | in |
| cg09867343 | 0.00744 | 0.00079 | 0.985 | LOC652276 | inside intron | 11554 | hyper | in |
| cg24283375 | 0.01116 | 0.00080 | 0.985 | ST3GAL3 | inside intron | 24666 | hyper | in |
| cg27190014 | 0.01448 | 0.00080 | 0.985 | PVT1 | inside intron | 68636 | hyper | in |
| cg13574488 | 0.01088 | 0.00081 | 0.985 | EMX2 | inside intron | 4290 | hyper | in |
| cg15304012 | 0.02043 | 0.00081 | 0.985 | RPS6KA2 | inside intron | 399281 | hyper | in |
| cg00071692 | 0.01433 | 0.00082 | 0.985 | SFI1 | inside intron | 37756 | hyper | in |
| cg24058566 | 0.00892 | 0.00082 | 0.985 | LINC00461 | inside intron | 162 | hyper | in |
| cg24898675 | 0.01594 | 0.00082 | 0.985 | SMAD3 | inside intron | 32199 | hyper | in |
| cg20812052 | 0.00707 | 0.00082 | 0.985 | PTDSS2 | inside intron | 27581 | hyper | in |
| cg06547959 | 0.00892 | 0.00083 | 0.985 | CELF4 | inside intron | 172010 | hyper | in |
| cg21287054 | 0.04104 | 0.00083 | 0.985 | TTYH1 | inside intron | 1407 | hyper | in |
| cg04694615 | 0.01342 | 0.00083 | 0.985 | NUP210 | inside intron | 40861 | hyper | in |
| cg19295951 | 0.01044 | 0.00084 | 0.985 | DUOXA1 | inside intron | 77 | hyper | in |
| cg13126552 | 0.02579 | 0.00084 | 0.985 | DNAH1 | inside intron | 1264 | hyper | in |
| cg23708673 | 0.01337 | 0.00086 | 0.985 | ATP2A2 | inside intron | 35474 | hyper | in |
| cg14634738 | 0.02856 | 0.00086 | 0.985 | RBM6 | inside intron | 148623 | hyper | in |
| cg00424286 | 0.03469 | 0.00087 | 0.985 | RPS6KA2 | inside intron | 387892 | hyper | in |
| cg22729960 | 0.00894 | 0.00087 | 0.985 | PTPRU | inside intron | 47397 | hyper | in |
| cg06103395 | 0.01335 | 0.00087 | 0.985 | TRERF1 | inside intron | 24079 | hyper | in |
| cg15957050 | 0.04377 | 0.00088 | 0.985 | RAPGEF3 | inside intron | 1365 | hyper | in |
| cg16092310 | 0.01053 | 0.00088 | 0.985 | DAND5 | inside intron | 620 | hyper | in |
| cg19764668 | 0.03602 | 0.00088 | 0.985 | SP110 | inside intron | 22675 | hyper | in |
| cg21107223 | 0.00358 | 0.00089 | 0.985 | MPI | inside intron | 45 | hyper | in |
| cg15850878 | 0.00983 | 0.00089 | 0.985 | DGKD | inside intron | 25470 | hyper | in |
| cg06408459 | 0.05750 | 0.00090 | 0.985 | PRKN | inside intron | 1353650 | hyper | in |
| cg03739378 | 0.00768 | 0.00090 | 0.985 | ATXN7L1 | inside intron | 22605 | hyper | in |
| cg18618113 | 0.01474 | 0.00090 | 0.985 | MITF | inside intron | 43215 | hyper | in |
| cg00898478 | 0.01200 | 0.00090 | 0.985 | PTCH1 | inside intron | 6464 | hyper | in |
| cg20386705 | 0.00546 | 0.00090 | 0.985 | BTBD9 | inside intron | 629 | hyper | in |
| cg14372524 | 0.03691 | 0.00091 | 0.985 | GLIS1 | inside intron | 36017 | hyper | in |
| cg20352386 | 0.03287 | 0.00092 | 0.985 | FBRSL1 | inside intron | 16436 | hyper | in |
| cg00002749 | 0.00560 | 0.00093 | 0.985 | VMP1 | inside intron | 54675 | hyper | in |
| cg19322788 | 0.00883 | 0.00093 | 0.985 | TMEM132B | inside intron | 20005 | hyper | in |
| cg07842386 | 0.01878 | 0.00094 | 0.985 | IL23R | inside intron | 27438 | hyper | in |
| cg19771773 | 0.00578 | 0.00095 | 0.985 | CARD8 | inside intron | 45587 | hyper | in |
| cg13568258 | 0.00895 | 0.00095 | 0.985 | CCDC114 | inside intron | 1764 | hyper | in |
| cg25846723 | 0.01714 | 0.00095 | 0.985 | ATP10A | inside intron | 1369 | hyper | in |
| cg11306701 | 0.01514 | 0.00095 | 0.985 | CTSL | inside intron | 484 | hyper | in |
| cg26571193 | 0.01264 | 0.00096 | 0.985 | FAM110A | inside intron | 4610 | hyper | in |
| cg22581896 | 0.02222 | 0.00096 | 0.985 | FOXK1 | inside intron | 57295 | hyper | in |
| cg23928982 | 0.01511 | 0.00096 | 0.985 | AKT1 | inside intron | 22038 | hyper | in |
| cg02341310 | 0.00211 | 0.00096 | 0.985 | TXLNB | inside intron | 21797 | hyper | in |
| cg14347088 | 0.01955 | 0.00096 | 0.985 | LRFN5 | inside intron | 2137 | hyper | in |
| cg16734549 | 0.01832 | 0.00097 | 0.985 | ARHGAP27 | inside intron | 14461 | hyper | in |
| cg07867072 | 0.01938 | 0.00098 | 0.985 | NEB | inside intron | 83117 | hyper | in |
| cg13480228 | 0.02788 | 0.00098 | 0.985 | MYO10 | inside intron | 129368 | hyper | in |
| cg13485685 | 0.01204 | 0.00099 | 0.985 | SLIT2 | inside intron | 570 | hyper | in |
| cg08984500 | 0.00601 | 0.00100 | 0.985 | DPEP1 | inside intron | 5422 | hyper | in |
| cg07000467 | -0.00407 | 0.00002 | 0.776 | DAB2 | inside exon | 405 | hypo | in |
| cg21809744 | -0.01279 | 0.00005 | 0.985 | ZNF837 | inside exon | 11756 | hypo | in |
| cg02036827 | -0.00316 | 0.00008 | 0.985 | FBXO46 | inside exon | 18547 | hypo | in |
| cg13922111 | -0.00257 | 0.00013 | 0.985 | ABHD13 | inside exon | 37 | hypo | in |
| cg01206970 | -0.00728 | 0.00015 | 0.985 | LIN7B | inside exon | 3569 | hypo | in |
| cg07933493 | -0.01625 | 0.00020 | 0.985 | RDH16 | inside exon | 5484 | hypo | in |
| cg11824369 | -0.00053 | 0.00026 | 0.985 | SIRT5 | inside exon | 4 | hypo | in |
| cg27304199 | -0.00975 | 0.00035 | 0.985 | MRPS18B | inside exon | 2028 | hypo | in |
| cg27461800 | -0.00260 | 0.00050 | 0.985 | KDM5A | inside exon | 266 | hypo | in |
| cg19763025 | -0.01752 | 0.00054 | 0.985 | LAMB1 | inside exon | 66197 | hypo | in |
| cg07955541 | -0.00497 | 0.00055 | 0.985 | WDR60 | inside exon | 39148 | hypo | in |
| cg25308542 | -0.00032 | 0.00056 | 0.985 | MIR17HG | inside exon | 39 | hypo | in |
| cg20887442 | -0.01508 | 0.00059 | 0.985 | NUMA1 | inside exon | 66096 | hypo | in |
| cg05003890 | -0.00885 | 0.00064 | 0.985 | MYOM2 | inside exon | 53594 | hypo | in |
| cg24852756 | -0.00299 | 0.00074 | 0.985 | CRTC1 | inside exon | 202 | hypo | in |
| cg05915481 | -0.00129 | 0.00076 | 0.985 | TARS | inside exon | 40 | hypo | in |
| cg01554235 | -0.00180 | 0.00088 | 0.985 | NME7 | inside exon | 143 | hypo | in |
| cg05655106 | -0.00896 | 0.00094 | 0.985 | AHRR | inside exon | 31203 | hypo | in |
| cg04900999 | -0.00139 | 0.00098 | 0.985 | ATP6V0A1 | inside exon | 96 | hypo | in |
| cg14193825 | -0.01252 | 0.00005 | 0.939 | NXN | inside intron | 77474 | hypo | in |
| cg23045373 | -0.00226 | 0.00006 | 0.985 | FAM167A | inside intron | 228 | hypo | in |
| cg22584104 | -0.02924 | 0.00009 | 0.985 | GALNT18 | inside intron | 63327 | hypo | in |
| cg11855842 | -0.01971 | 0.00022 | 0.985 | ASAP3 | inside intron | 3565 | hypo | in |
| cg11293828 | -0.03687 | 0.00027 | 0.985 | PRMT8 | inside intron | 107820 | hypo | in |
| cg19304088 | -0.01586 | 0.00027 | 0.985 | PITX2 | inside intron | 15489 | hypo | in |
| cg21199251 | -0.01164 | 0.00028 | 0.985 | AJAP1 | inside intron | 113516 | hypo | in |
| cg22760037 | -0.01065 | 0.00031 | 0.985 | LINC00673 | inside intron | 170779 | hypo | in |
| cg00771778 | -0.00919 | 0.00031 | 0.985 | LYST | inside intron | 31513 | hypo | in |
| cg11709728 | -0.00549 | 0.00038 | 0.985 | LRRC1 | inside intron | 84396 | hypo | in |
| cg17461336 | -0.02095 | 0.00038 | 0.985 | CYP3A43 | inside intron | 15923 | hypo | in |
| cg17011300 | -0.00985 | 0.00041 | 0.985 | ESRRG | inside intron | 50267 | hypo | in |
| cg21079591 | -0.01771 | 0.00042 | 0.985 | ATP6V1G2-DDX39B | inside intron | 3890 | hypo | in |
| cg18908374 | -0.00751 | 0.00043 | 0.985 | SHANK2 | inside intron | 21731 | hypo | in |
| cg21758687 | -0.00232 | 0.00043 | 0.985 | PIAS1 | inside intron | 155 | hypo | in |
| cg10982668 | -0.00369 | 0.00048 | 0.985 | INMT | inside intron | 35822 | hypo | in |
| cg02859962 | -0.01410 | 0.00049 | 0.985 | LINC00284 | inside intron | 2394 | hypo | in |
| cg12678623 | -0.00208 | 0.00054 | 0.985 | ATP5MG | inside intron | 699 | hypo | in |
| cg06567964 | -0.00413 | 0.00059 | 0.985 | SREK1IP1 | inside intron | 630 | hypo | in |
| cg03956606 | -0.00408 | 0.00064 | 0.985 | MTOR | inside intron | 73551 | hypo | in |
| cg14210634 | -0.00184 | 0.00067 | 0.985 | LDLRAP1 | inside intron | 215 | hypo | in |
| cg21936550 | -0.00382 | 0.00068 | 0.985 | TBL2 | inside intron | 291 | hypo | in |
| cg04012872 | -0.01047 | 0.00070 | 0.985 | IPP | inside intron | 3712 | hypo | in |
| cg16062253 | -0.01198 | 0.00071 | 0.985 | NOTCH4 | inside intron | 21350 | hypo | in |
| cg05384198 | -0.00588 | 0.00072 | 0.985 | TBC1D2 | inside intron | 1812 | hypo | in |
| cg14963283 | -0.00793 | 0.00075 | 0.985 | CYFIP1 | inside intron | 5881 | hypo | in |
| cg19270965 | -0.00476 | 0.00075 | 0.985 | EZH1 | inside intron | 40083 | hypo | in |
| cg06673490 | -0.00923 | 0.00078 | 0.985 | GNG4 | inside intron | 61129 | hypo | in |
| cg25125469 | -0.04606 | 0.00084 | 0.985 | ACSM3 | inside intron | 3103 | hypo | in |
| cg00203456 | -0.00151 | 0.00085 | 0.985 | ZSCAN16-AS1 | inside intron | 12724 | hypo | in |
| cg21131421 | -0.01667 | 0.00087 | 0.985 | RAD51B | inside intron | 630719 | hypo | in |
| cg27492749 | -0.00205 | 0.00089 | 0.985 | SGCE | inside intron | 621 | hypo | in |
| cg08296556 | -0.00067 | 0.00090 | 0.985 | PHTF2 | inside intron | 552 | hypo | in |
| cg16893389 | -0.01237 | 0.00090 | 0.985 | FOXRED1 | inside intron | 4636 | hypo | in |
| cg01595204 | -0.00288 | 0.00091 | 0.985 | TCF3 | inside intron | 9491 | hypo | in |
| cg02555727 | -0.01178 | 0.00098 | 0.985 | SLC15A4 | inside intron | 26995 | hypo | in |
| cg12617928 | 0.02148 | 0.00012 | 0.985 | HBQ1 | close to 3' | 1409 | hyper | out |
| cg00602811 | 0.04467 | 0.00036 | 0.985 | ZEB2-AS1 | close to 3' | 1383 | hyper | out |
| cg05397074 | 0.01507 | 0.00043 | 0.985 | MIR4493 | close to 3' | 139 | hyper | out |
| cg16746471 | 0.01607 | 0.00001 | 0.688 | KIAA1324L | promoter | 374 | hyper | out |
| cg27208052 | 0.02457 | 0.00002 | 0.776 | MMP15 | promoter | 640 | hyper | out |
| cg20284913 | 0.00656 | 0.00003 | 0.885 | RFX1 | promoter | 109 | hyper | out |
| cg27402569 | 0.00566 | 0.00004 | 0.885 | ZMYM4 | promoter | 228 | hyper | out |
| cg21827892 | 0.03907 | 0.00005 | 0.939 | SLC7A9 | promoter | 360 | hyper | out |
| cg25424659 | 0.01596 | 0.00006 | 0.985 | RAB3D | promoter | 248 | hyper | out |
| cg04730882 | 0.00796 | 0.00009 | 0.985 | HOXD9 | promoter | 878 | hyper | out |
| cg14970695 | 0.04617 | 0.00010 | 0.985 | PTCHD3P1 | promoter | 36 | hyper | out |
| cg16213655 | 0.00900 | 0.00013 | 0.985 | PPL | promoter | 396 | hyper | out |
| cg06275875 | 0.01310 | 0.00013 | 0.985 | CTU1 | promoter | 690 | hyper | out |
| cg03363242 | 0.00692 | 0.00021 | 0.985 | INTS9 | promoter | 40 | hyper | out |
| cg20478750 | 0.01999 | 0.00023 | 0.985 | SH3RF2 | promoter | 842 | hyper | out |
| cg11539780 | 0.01048 | 0.00023 | 0.985 | ZNF471 | promoter | 196 | hyper | out |
| cg24453493 | 0.02450 | 0.00024 | 0.985 | ZNF503-AS1 | promoter | 6 | hyper | out |
| cg04117547 | 0.01362 | 0.00027 | 0.985 | C9orf47 | promoter | 2344 | hyper | out |
| cg00770085 | 0.01128 | 0.00028 | 0.985 | TTC39A | promoter | 51 | hyper | out |
| cg03381598 | 0.02046 | 0.00028 | 0.985 | GRM7 | promoter | 702 | hyper | out |
| cg25564272 | 0.01155 | 0.00029 | 0.985 | TTLL6 | promoter | 1679 | hyper | out |
| cg16565993 | 0.00731 | 0.00029 | 0.985 | GNA14 | promoter | 649 | hyper | out |
| cg03727732 | 0.01076 | 0.00031 | 0.985 | CLK3 | promoter | 465 | hyper | out |
| cg12465710 | 0.03116 | 0.00031 | 0.985 | UTF1 | promoter | 893 | hyper | out |
| cg17476312 | 0.02941 | 0.00032 | 0.985 | CS | promoter | 614 | hyper | out |
| cg17252960 | 0.01075 | 0.00032 | 0.985 | ID4 | promoter | 251 | hyper | out |
| cg06667339 | 0.01392 | 0.00033 | 0.985 | KIF5A | promoter | 450 | hyper | out |
| cg18643762 | 0.00784 | 0.00034 | 0.985 | STEAP3 | promoter | 212 | hyper | out |
| cg24624629 | 0.04316 | 0.00034 | 0.985 | KRTAP2-1 | promoter | 53 | hyper | out |
| cg05771342 | 0.05300 | 0.00034 | 0.985 | MRGPRF | promoter | 1352 | hyper | out |
| cg24112562 | 0.00187 | 0.00036 | 0.985 | SIM2 | promoter | 1950 | hyper | out |
| cg24621354 | 0.00987 | 0.00039 | 0.985 | TES | promoter | 165 | hyper | out |
| cg10085651 | 0.00993 | 0.00039 | 0.985 | PSMD7 | promoter | 437 | hyper | out |
| cg21007783 | 0.04543 | 0.00042 | 0.985 | SPIB | promoter | 14 | hyper | out |
| cg13322582 | 0.01270 | 0.00042 | 0.985 | DPF1 | promoter | 372 | hyper | out |
| cg02514528 | 0.00992 | 0.00043 | 0.985 | Mar-02 | promoter | 4 | hyper | out |
| cg23355952 | 0.00643 | 0.00045 | 0.985 | TEPSIN | promoter | 41 | hyper | out |
| cg10397322 | 0.02346 | 0.00045 | 0.985 | UBE2E2 | promoter | 602 | hyper | out |
| cg02652597 | 0.02374 | 0.00046 | 0.985 | VAMP5 | promoter | 239 | hyper | out |
| cg12648537 | 0.02595 | 0.00046 | 0.985 | RGS22 | promoter | 119 | hyper | out |
| cg23736485 | 0.00918 | 0.00048 | 0.985 | WIZ | promoter | 202 | hyper | out |
| cg16328342 | 0.01140 | 0.00049 | 0.985 | MSX2 | promoter | 6 | hyper | out |
| cg26608174 | 0.02987 | 0.00056 | 0.985 | MIR196A1 | promoter | 1114 | hyper | out |
| cg24533810 | 0.01279 | 0.00058 | 0.985 | GDNF | promoter | 134 | hyper | out |
| cg23744909 | 0.00727 | 0.00058 | 0.985 | ZFAND4 | promoter | 203 | hyper | out |
| cg10912268 | 0.00439 | 0.00059 | 0.985 | SPTLC1 | promoter | 212 | hyper | out |
| cg02990869 | 0.01279 | 0.00059 | 0.985 | Mar-01 | promoter | 435 | hyper | out |
| cg21269843 | 0.00915 | 0.00059 | 0.985 | OTP | promoter | 174 | hyper | out |
| cg12325311 | 0.03477 | 0.00060 | 0.985 | NCCRP1 | promoter | 1669 | hyper | out |
| cg25187161 | 0.05176 | 0.00060 | 0.985 | SRSF4 | promoter | 1388 | hyper | out |
| cg01768342 | 0.00846 | 0.00061 | 0.985 | CATSPER4 | promoter | 63 | hyper | out |
| cg04905434 | 0.00739 | 0.00063 | 0.985 | MAFA | promoter | 171 | hyper | out |
| cg02664349 | 0.00793 | 0.00066 | 0.985 | PIEZO2 | promoter | 1080 | hyper | out |
| cg16162391 | 0.04387 | 0.00068 | 0.985 | PTCHD3P1 | promoter | 115 | hyper | out |
| cg23670203 | 0.01113 | 0.00068 | 0.985 | STK38 | promoter | 609 | hyper | out |
| cg03699904 | 0.02116 | 0.00069 | 0.985 | SLC2A2 | promoter | 1261 | hyper | out |
| cg20743974 | 0.01321 | 0.00070 | 0.985 | LIN9 | promoter | 520 | hyper | out |
| cg19541444 | 0.00402 | 0.00071 | 0.985 | RAB6A | promoter | 4 | hyper | out |
| cg20772512 | 0.03592 | 0.00071 | 0.985 | DNALI1 | promoter | 161 | hyper | out |
| cg06758407 | 0.00302 | 0.00072 | 0.985 | TOB2 | promoter | 170 | hyper | out |
| cg16658020 | 0.02000 | 0.00073 | 0.985 | KRTAP2-1 | promoter | 73 | hyper | out |
| cg09866366 | 0.02716 | 0.00074 | 0.985 | ABCF3 | promoter | 548 | hyper | out |
| cg13468002 | 0.01855 | 0.00075 | 0.985 | CAMKV | promoter | 99 | hyper | out |
| cg21497218 | 0.00515 | 0.00078 | 0.985 | GRN | promoter | 107 | hyper | out |
| cg14400389 | 0.00405 | 0.00078 | 0.985 | NUP85 | promoter | 270 | hyper | out |
| cg18341059 | 0.00340 | 0.00087 | 0.985 | AHNAK | promoter | 175 | hyper | out |
| cg16541522 | 0.00450 | 0.00090 | 0.985 | DUS3L | promoter | 99 | hyper | out |
| cg17804112 | 0.03060 | 0.00091 | 0.985 | NCR1 | promoter | 12 | hyper | out |
| cg15237923 | 0.01260 | 0.00092 | 0.985 | CSMD1 | promoter | 96 | hyper | out |
| cg03496157 | 0.01524 | 0.00094 | 0.985 | BCL7A | promoter | 968 | hyper | out |
| cg11115427 | 0.02404 | 0.00094 | 0.985 | PHYH | promoter | 480 | hyper | out |
| cg18257541 | 0.01122 | 0.00096 | 0.985 | UBE2E2 | promoter | 591 | hyper | out |
| cg07263235 | 0.02188 | 0.00098 | 0.985 | LPL | promoter | 188 | hyper | out |
| cg22518632 | 0.01389 | 0.00099 | 0.985 | CSMD3 | promoter | 233 | hyper | out |
| cg08283932 | 0.00184 | 0.00099 | 0.985 | ADA | promoter | 331 | hyper | out |
| cg25506396 | 0.00364 | 0.00099 | 0.985 | DGKD | promoter | 286 | hyper | out |
| cg00362604 | 0.01582 | 0.00099 | 0.985 | SAYSD1 | promoter | 329 | hyper | out |
| cg12322720 | 0.05508 | 0.00001 | 0.688 | FOXB1 | downstream | 150921 | hyper | out |
| cg06955687 | 0.03721 | 0.00001 | 0.688 | DDX25 | downstream | 28769 | hyper | out |
| ch.22.707049R | 0.01251 | 0.00001 | 0.688 | TNRC6B | downstream | 159737 | hyper | out |
| cg09344183 | 0.01437 | 0.00001 | 0.688 | SP9 | downstream | 5964 | hyper | out |
| cg16514411 | 0.02231 | 0.00003 | 0.776 | COL20A1 | downstream | 29627 | hyper | out |
| cg15793577 | 0.01768 | 0.00006 | 0.985 | PSMB1 | downstream | 114674 | hyper | out |
| cg21323720 | 0.05601 | 0.00007 | 0.985 | CAMK2D | downstream | 325482 | hyper | out |
| cg06878681 | 0.00853 | 0.00007 | 0.985 | HSD17B3 | downstream | 86357 | hyper | out |
| cg15715743 | 0.02142 | 0.00008 | 0.985 | IKZF4 | downstream | 13805 | hyper | out |
| cg18049969 | 0.02942 | 0.00012 | 0.985 | DBI | downstream | 31315 | hyper | out |
| cg16399625 | 0.01101 | 0.00012 | 0.985 | ACTN1 | downstream | 123895 | hyper | out |
| cg08594650 | 0.04071 | 0.00014 | 0.985 | ACTR3B | downstream | 151039 | hyper | out |
| cg09644974 | 0.01997 | 0.00023 | 0.985 | MIR1246 | downstream | 107642 | hyper | out |
| cg10785622 | 0.01517 | 0.00024 | 0.985 | NKX6-1 | downstream | 15978 | hyper | out |
| cg08792388 | 0.02227 | 0.00026 | 0.985 | CCR6 | downstream | 24468 | hyper | out |
| cg27664085 | 0.08228 | 0.00026 | 0.985 | MIR7-2 | downstream | 2759 | hyper | out |
| cg25527770 | 0.01544 | 0.00028 | 0.985 | RPS15 | downstream | 6529 | hyper | out |
| cg03426023 | 0.01156 | 0.00031 | 0.985 | IRX5 | downstream | 6094 | hyper | out |
| cg08367035 | 0.00727 | 0.00033 | 0.985 | SORL1 | downstream | 132494 | hyper | out |
| cg16658180 | 0.01546 | 0.00037 | 0.985 | SERTAD4 | downstream | 14945 | hyper | out |
| cg00676711 | 0.01094 | 0.00039 | 0.985 | GFRA2 | downstream | 378004 | hyper | out |
| cg26963367 | 0.08216 | 0.00040 | 0.985 | MIR7-2 | downstream | 2785 | hyper | out |
| cg08591023 | 0.01918 | 0.00041 | 0.985 | VSTM1 | downstream | 27914 | hyper | out |
| cg03008525 | 0.00573 | 0.00043 | 0.985 | NRTN | downstream | 5597 | hyper | out |
| cg27082433 | 0.01744 | 0.00045 | 0.985 | TRAF1 | downstream | 34332 | hyper | out |
| ch.10.80205838R | 0.01014 | 0.00046 | 0.985 | ZMIZ1-AS1 | downstream | 291373 | hyper | out |
| cg22542050 | 0.02854 | 0.00046 | 0.985 | LINC01776 | downstream | 248244 | hyper | out |
| cg23923204 | 0.04011 | 0.00052 | 0.985 | TPBGL | downstream | 7389 | hyper | out |
| cg05883620 | 0.01629 | 0.00053 | 0.985 | WDFY3-AS2 | downstream | 118455 | hyper | out |
| cg09588555 | 0.01064 | 0.00054 | 0.985 | EDN2 | downstream | 102676 | hyper | out |
| cg15014586 | 0.01098 | 0.00055 | 0.985 | LINC00941 | downstream | 48980 | hyper | out |
| cg15852141 | 0.01231 | 0.00057 | 0.985 | DAD1 | downstream | 166760 | hyper | out |
| cg08084219 | 0.02338 | 0.00059 | 0.985 | ADCY9 | downstream | 169812 | hyper | out |
| cg03504160 | 0.01579 | 0.00063 | 0.985 | MRFAP1 | downstream | 17376 | hyper | out |
| cg19965949 | 0.01231 | 0.00064 | 0.985 | CCSER2 | downstream | 502549 | hyper | out |
| cg19624780 | 0.01462 | 0.00065 | 0.985 | ARHGAP12 | downstream | 231431 | hyper | out |
| cg15868235 | 0.03243 | 0.00066 | 0.985 | PPARG | downstream | 93113 | hyper | out |
| cg05637886 | 0.01553 | 0.00069 | 0.985 | RMI2 | downstream | 14371 | hyper | out |
| cg09982347 | 0.03056 | 0.00070 | 0.985 | SPTSSA | downstream | 105912 | hyper | out |
| cg17752901 | 0.02354 | 0.00077 | 0.985 | SRSF6 | downstream | 14531 | hyper | out |
| cg15043935 | 0.01927 | 0.00084 | 0.985 | GADD45B | downstream | 11696 | hyper | out |
| cg08687661 | 0.00917 | 0.00087 | 0.985 | KAZALD1 | downstream | 12628 | hyper | out |
| cg17373555 | 0.01605 | 0.00088 | 0.985 | MYO18A | downstream | 109670 | hyper | out |
| cg20687616 | 0.00630 | 0.00095 | 0.985 | PLPP3 | downstream | 161521 | hyper | out |
| cg15645372 | 0.01751 | 0.00095 | 0.985 | SOST | downstream | 41430 | hyper | out |
| cg24060040 | 0.02920 | 0.00001 | 0.629 | DUS3L | upstream | 11018 | hyper | out |
| cg07311956 | 0.04950 | 0.00004 | 0.885 | PTPN2 | upstream | 26693 | hyper | out |
| cg14337167 | 0.00853 | 0.00008 | 0.985 | WNT4 | upstream | 20582 | hyper | out |
| cg14459158 | 0.05356 | 0.00008 | 0.985 | BARX1 | upstream | 2954 | hyper | out |
| cg26444345 | 0.04331 | 0.00011 | 0.985 | CAPN2 | upstream | 12264 | hyper | out |
| cg00928167 | 0.02582 | 0.00011 | 0.985 | NKX2-1 | upstream | 4586 | hyper | out |
| cg05854154 | 0.03542 | 0.00011 | 0.985 | ADGRL1 | upstream | 15354 | hyper | out |
| cg06936869 | 0.01222 | 0.00016 | 0.985 | TBX18 | upstream | 252087 | hyper | out |
| cg13834112 | 0.03303 | 0.00020 | 0.985 | ANPEP | upstream | 3567 | hyper | out |
| cg14930963 | 0.01271 | 0.00021 | 0.985 | PTPN1 | upstream | 61321 | hyper | out |
| cg16614139 | 0.02279 | 0.00025 | 0.985 | CREB1 | upstream | 27829 | hyper | out |
| cg22607981 | 0.02220 | 0.00027 | 0.985 | WIPF1 | upstream | 5657 | hyper | out |
| cg01309428 | 0.02744 | 0.00027 | 0.985 | COL12A1 | upstream | 11515 | hyper | out |
| cg18793404 | 0.04420 | 0.00032 | 0.985 | COBL | upstream | 771974 | hyper | out |
| cg13990926 | 0.01750 | 0.00034 | 0.985 | CALCOCO1 | upstream | 12140 | hyper | out |
| cg08863620 | 0.01951 | 0.00036 | 0.985 | GRID1 | upstream | 10518 | hyper | out |
| cg04071225 | 0.04552 | 0.00037 | 0.985 | SLC35G2 | upstream | 31837 | hyper | out |
| ch.6.15091466F | 0.01746 | 0.00039 | 0.985 | JARID2 | upstream | 262247 | hyper | out |
| cg16204818 | 0.00436 | 0.00045 | 0.985 | TMEM106B | upstream | 99492 | hyper | out |
| cg26736812 | 0.01025 | 0.00047 | 0.985 | DLX4 | upstream | 4209 | hyper | out |
| cg12603039 | 0.01303 | 0.00048 | 0.985 | FAM163A | upstream | 18223 | hyper | out |
| cg00948749 | 0.02645 | 0.00051 | 0.985 | NEURL1B | upstream | 82145 | hyper | out |
| cg03287519 | 0.05753 | 0.00055 | 0.985 | OXR1 | upstream | 50752 | hyper | out |
| cg18031880 | 0.03908 | 0.00055 | 0.985 | LINC00900 | upstream | 198587 | hyper | out |
| cg04112567 | 0.00738 | 0.00055 | 0.985 | MIR34A | upstream | 30504 | hyper | out |
| cg16362863 | 0.01794 | 0.00059 | 0.985 | MSI1 | upstream | 19704 | hyper | out |
| cg09349604 | 0.00963 | 0.00068 | 0.985 | TMEM33 | upstream | 56390 | hyper | out |
| cg08863473 | 0.01710 | 0.00068 | 0.985 | MIR4472-1 | upstream | 95300 | hyper | out |
| cg23667554 | 0.02269 | 0.00068 | 0.985 | NAPG | upstream | 5852 | hyper | out |
| cg06326646 | 0.02224 | 0.00068 | 0.985 | INA | upstream | 11274 | hyper | out |
| cg16863619 | 0.02185 | 0.00070 | 0.985 | PIK3R1 | upstream | 28339 | hyper | out |
| cg07614295 | 0.00710 | 0.00073 | 0.985 | BRD7 | upstream | 25775 | hyper | out |
| cg06832677 | 0.03817 | 0.00074 | 0.985 | STEAP3 | upstream | 4620 | hyper | out |
| cg14637762 | 0.00988 | 0.00075 | 0.985 | HAO2 | upstream | 82009 | hyper | out |
| cg10062327 | 0.03015 | 0.00082 | 0.985 | MIR3977 | upstream | 166502 | hyper | out |
| cg02893529 | 0.01294 | 0.00093 | 0.985 | RAI1 | upstream | 17891 | hyper | out |
| cg21181942 | 0.01620 | 0.00093 | 0.985 | ADRA2C | upstream | 41846 | hyper | out |
| cg01174600 | 0.02110 | 0.00094 | 0.985 | CD69 | upstream | 3486 | hyper | out |
| cg02246928 | 0.03643 | 0.00094 | 0.985 | UTRN | upstream | 20812 | hyper | out |
| cg03468466 | 0.00861 | 0.00100 | 0.985 | EOMES | upstream | 7708 | hyper | out |
| cg17316185 | -0.00354 | 0.00002 | 0.776 | C1QL1 | promoter | 240 | hypo | out |
| cg19148051 | -0.00376 | 0.00004 | 0.885 | LRRC47 | promoter | 28 | hypo | out |
| cg09596027 | -0.00984 | 0.00038 | 0.985 | DPRX | promoter | 599 | hypo | out |
| cg12131707 | -0.00845 | 0.00043 | 0.985 | CERS3 | promoter | 785 | hypo | out |
| cg22981461 | -0.03906 | 0.00046 | 0.985 | OTUB2 | promoter | 250 | hypo | out |
| cg10069827 | -0.01829 | 0.00050 | 0.985 | PTPN11 | promoter | 2167 | hypo | out |
| cg26685825 | -0.02724 | 0.00056 | 0.985 | KRTAP4-5 | promoter | 1447 | hypo | out |
| cg14918391 | -0.00208 | 0.00062 | 0.985 | CAPN5 | promoter | 111 | hypo | out |
| cg07673866 | -0.00601 | 0.00069 | 0.985 | KIF11 | promoter | 1449 | hypo | out |
| cg03807314 | -0.01508 | 0.00070 | 0.985 | MAP3K8 | promoter | 272 | hypo | out |
| cg14252136 | -0.01017 | 0.00074 | 0.985 | CYB5B | promoter | 853 | hypo | out |
| cg22241850 | -0.01558 | 0.00076 | 0.985 | NPC1L1 | promoter | 157 | hypo | out |
| cg14875384 | -0.00406 | 0.00089 | 0.985 | DMBX1 | promoter | 1024 | hypo | out |
| cg05177459 | -0.02831 | 0.00089 | 0.985 | MCOLN2 | promoter | 1494 | hypo | out |
| cg17684613 | -0.00562 | 0.00091 | 0.985 | OSBP2 | promoter | 341 | hypo | out |
| cg08577022 | -0.00857 | 0.00008 | 0.985 | ARSB | downstream | 268920 | hypo | out |
| cg16562127 | -0.00729 | 0.00008 | 0.985 | DLK1 | downstream | 42743 | hypo | out |
| cg15248809 | -0.00499 | 0.00010 | 0.985 | SHISA9 | downstream | 343223 | hypo | out |
| cg22856771 | -0.00997 | 0.00012 | 0.985 | SORL1 | downstream | 204708 | hypo | out |
| cg16274893 | -0.01067 | 0.00016 | 0.985 | RAD51B | downstream | 917584 | hypo | out |
| cg11499696 | -0.01344 | 0.00026 | 0.985 | EFNA5 | downstream | 795054 | hypo | out |
| cg09152136 | -0.01012 | 0.00042 | 0.985 | RASAL2 | downstream | 43172 | hypo | out |
| cg16514717 | -0.00705 | 0.00042 | 0.985 | NLN | downstream | 73771 | hypo | out |
| cg09473396 | -0.03174 | 0.00048 | 0.985 | ADAMTS9-AS2 | downstream | 461433 | hypo | out |
| cg06154159 | -0.01121 | 0.00049 | 0.985 | NA | downstream | 8567 | hypo | out |
| cg22547771 | -0.02227 | 0.00065 | 0.985 | LINC-ROR | downstream | 21542 | hypo | out |
| cg13258539 | -0.06536 | 0.00075 | 0.985 | TP53TG3D | downstream | 13570 | hypo | out |
| cg10866026 | -0.00545 | 0.00078 | 0.985 | LARP6 | downstream | 51810 | hypo | out |
| cg22829408 | -0.01019 | 0.00085 | 0.985 | YTHDF3 | downstream | 154329 | hypo | out |
| cg27019158 | -0.00825 | 0.00086 | 0.985 | DHRS4-AS1 | downstream | 126896 | hypo | out |
| cg27272098 | -0.02645 | 0.00089 | 0.985 | LINC01257 | downstream | 272326 | hypo | out |
| cg22445217 | -0.02885 | 0.00090 | 0.985 | QKI | downstream | 585401 | hypo | out |
| cg14444715 | -0.02419 | 0.00018 | 0.985 | MIR4307 | upstream | 35489 | hypo | out |
| cg05498047 | -0.00499 | 0.00028 | 0.985 | ROBO3 | upstream | 20403 | hypo | out |
| cg16844564 | -0.00794 | 0.00031 | 0.985 | FILIP1 | upstream | 38988 | hypo | out |
| cg14139242 | -0.01647 | 0.00038 | 0.985 | IRX4 | upstream | 120023 | hypo | out |
| cg04282705 | -0.01800 | 0.00038 | 0.985 | SEMA3A | upstream | 341610 | hypo | out |
| cg25125937 | -0.00442 | 0.00039 | 0.985 | ZFPM2 | upstream | 362809 | hypo | out |
| cg04520945 | -0.00486 | 0.00055 | 0.985 | ULBP3 | upstream | 11276 | hypo | out |
| cg09202928 | -0.00914 | 0.00068 | 0.985 | FBRSL1 | upstream | 22163 | hypo | out |
| cg01195278 | -0.00969 | 0.00072 | 0.985 | LINC00593 | upstream | 77783 | hypo | out |
| cg08426030 | -0.02342 | 0.00075 | 0.985 | KCNN2 | upstream | 343853 | hypo | out |
| cg12113013 | -0.01075 | 0.00082 | 0.985 | TCERG1L | upstream | 98957 | hypo | out |
| cg20919587 | -0.02331 | 0.00084 | 0.985 | TUBB8 | upstream | 38442 | hypo | out |
| cg23064873 | -0.01764 | 0.00096 | 0.985 | ZNF703 | upstream | 95728 | hypo | out |
| cg24736167 | -0.02688 | 0.00097 | 0.985 | OR10H2 | upstream | 10259 | hypo | out |


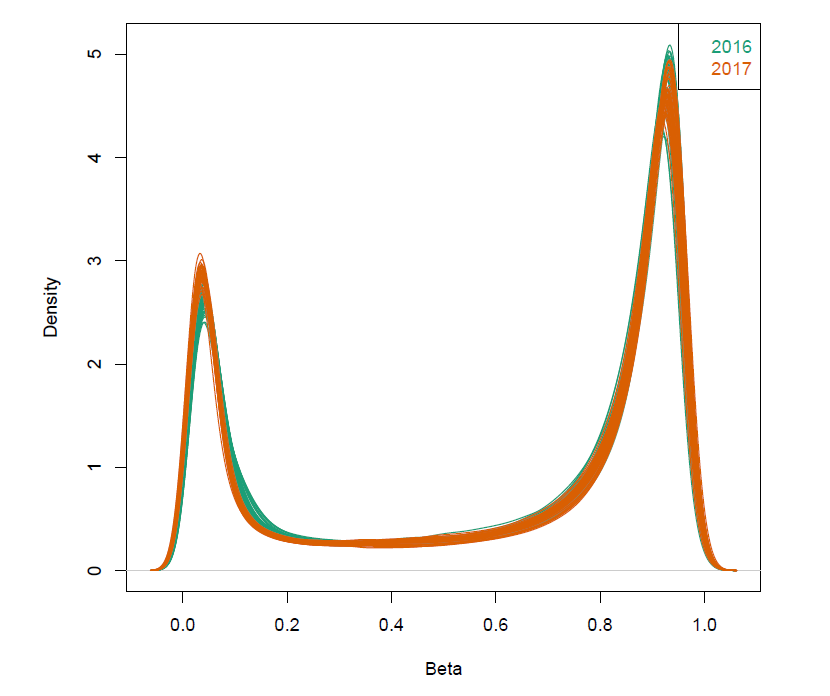


**Supplementary Figure 1. Preprocessing using noob normalisation.** The distribution of individuals as displayed as lines were plotted based on beta density post normalisation using noob. Data coloured based on year the measurements took place; 2016 (green) or 2017 (orange).


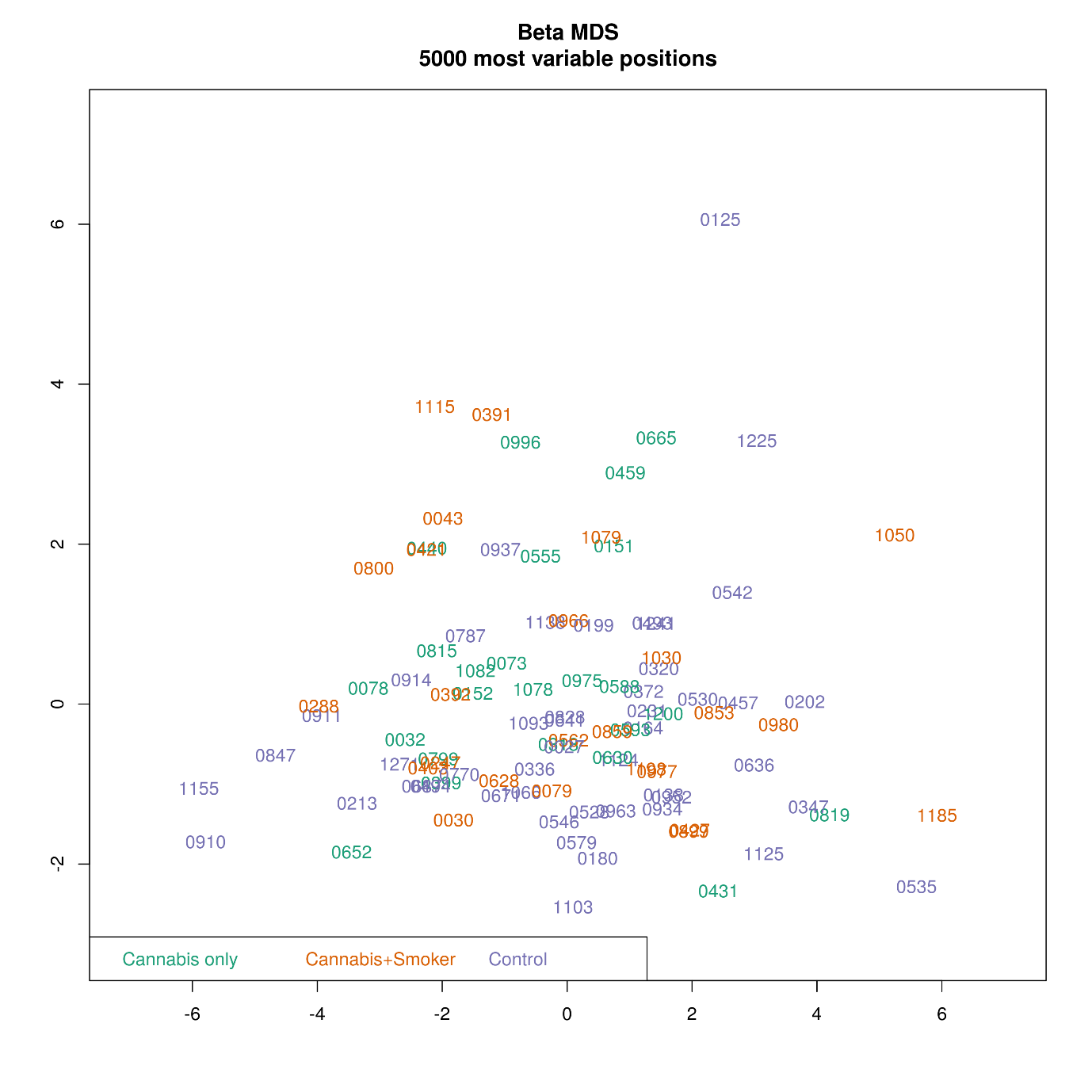


**Supplementary Figure 2. A multidimensional plot displaying the 5000 most variable positions post normalisation using noob.** Individuals are grouped in colour by status: cannabis-only users (green), cannabis with tobacco (orange) or control (purple).


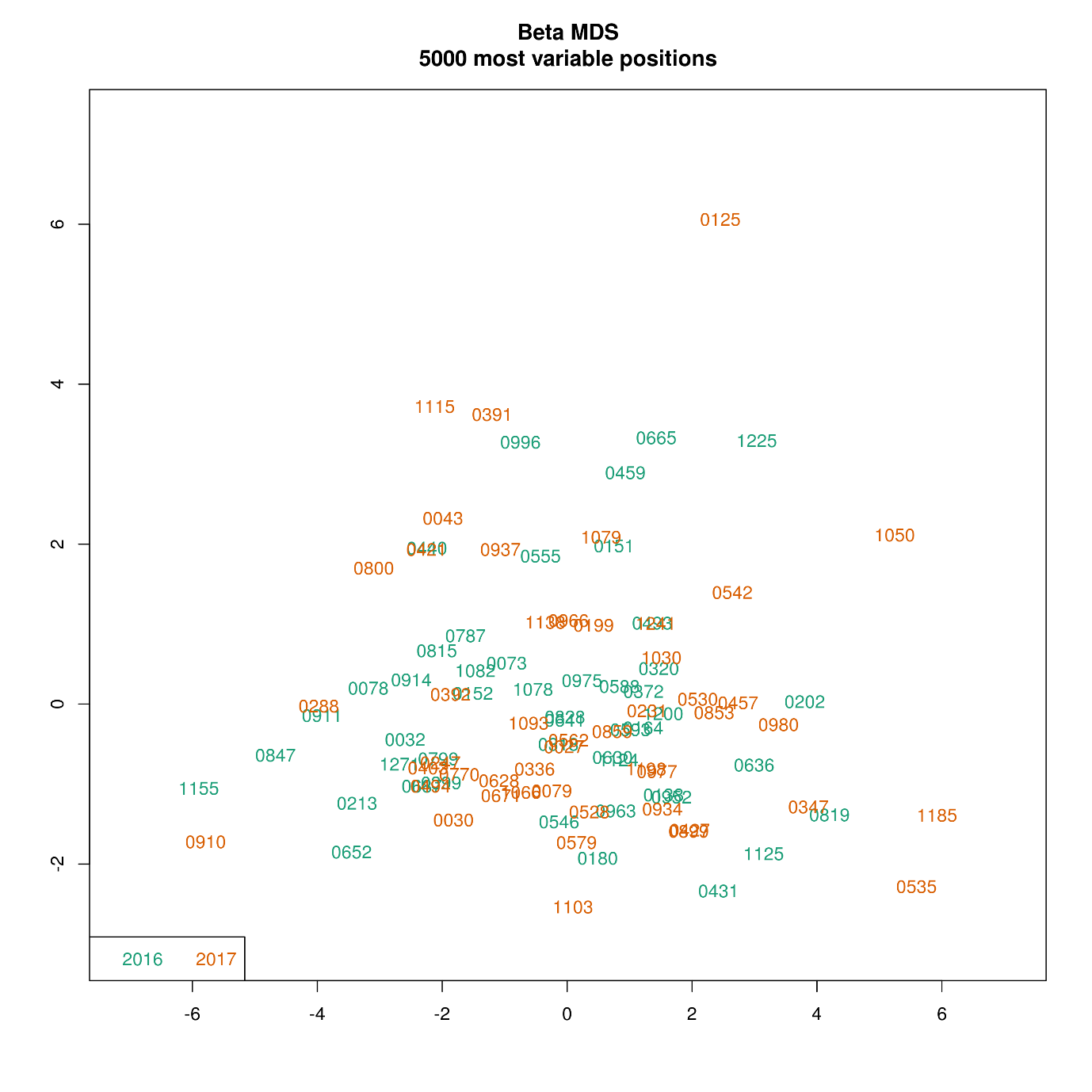


**Supplementary Figure 3. A multidimensional plot displaying the 5000 most variable positions post normalisation using noob.** Individuals are coloured on year the measurements were collected.

**
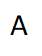
**

B


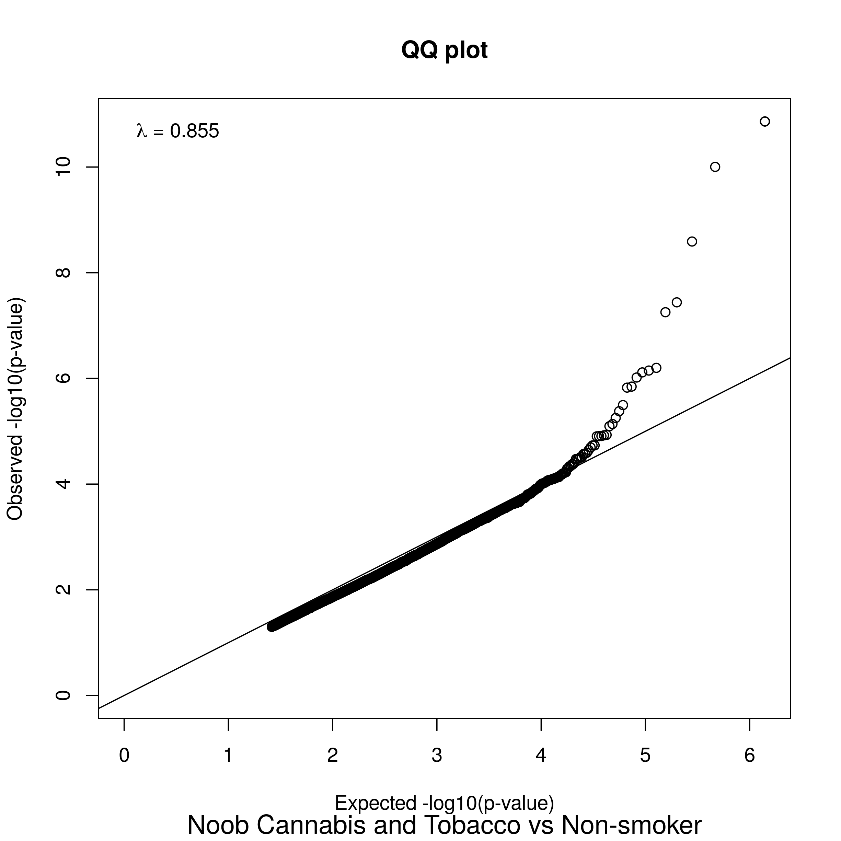

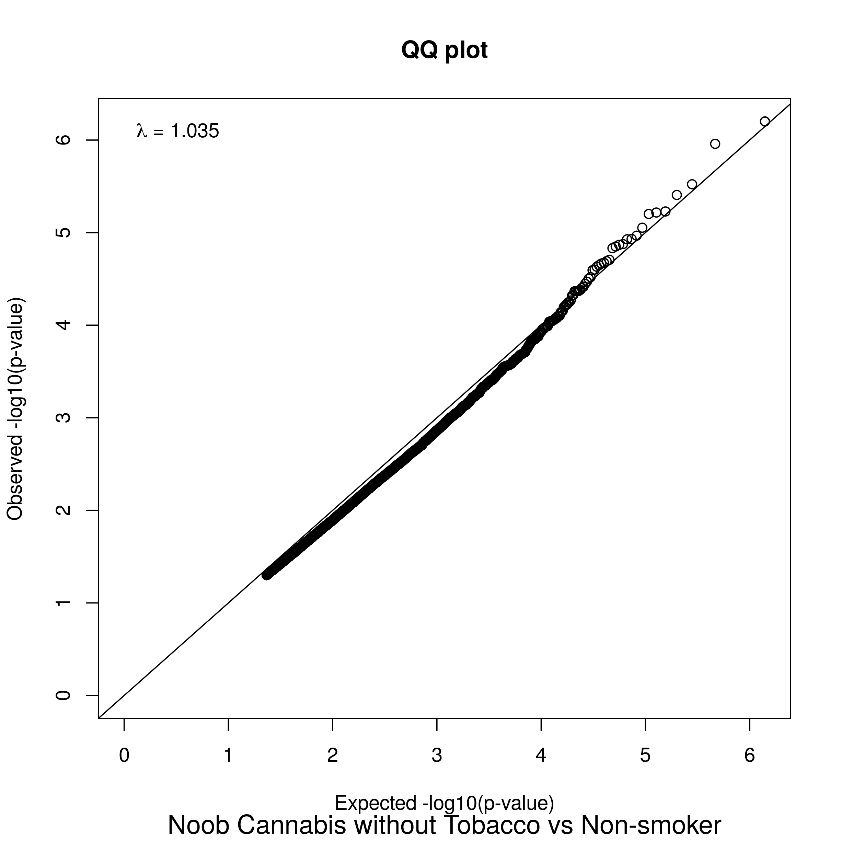


**Supplementary Figure 4. Quantile- quantile plots.** Quantile plots were used to assess for overfitting of models. A) Cannabis-only vs control samples B) Cannabis with tobacco vs control. Each dot displays the expected –log10 (p-values) under the model.
